# Antidepressants Differentially Regulate Intracellular Signaling from α1-Adrenergic Receptor Subtypes In Vitro

**DOI:** 10.3390/ijms22094817

**Published:** 2021-05-01

**Authors:** Piotr Chmielarz, Justyna Kuśmierczyk, Katarzyna Rafa-Zabłocka, Katarzyna Chorązka, Marta Kowalska, Grzegorz Satała, Irena Nalepa

**Affiliations:** 1 Department of Brain Biochemistry, Maj Institute of Pharmacology, Polish Academy of Sciences, Smętna 12, 31-343 Kraków, Poland; chmiel@if-pan.krakow.pl (P.C.); justyna.kusmierczyk@awf.krakow.pl (J.K.); zablocka@if-pan.krakow.pl (K.R.-Z.); chorazka.katarzyna@gmail.com (K.C.); marcik48@op.pl (M.K.); 2 Department of Medicinal Chemistry, Maj Institute of Pharmacology, Polish Academy of Sciences, Smętna 12, 31-343 Kraków, Poland; satala@if-pan.krakow.pl

**Keywords:** alpha1-adrenergic receptor subtypes, antidepressants, imipramine, desipramine, mianserin, citalopram, antagonist, second messenger, inositol phosphate, G-protein-coupled receptor

## Abstract

Currently utilized antidepressants have limited effectiveness and frequently incur undesired effects. Most antidepressants are thought to act via the inhibition of monoamine reuptake; however, direct binding to monoaminergic receptors has been proposed to contribute to both their clinical effectiveness and their side effects, or lack thereof. Among the target receptors of antidepressants, α1‑adrenergic receptors (ARs) have been implicated in depression etiology, antidepressant action, and side effects. However, differences in the direct effects of antidepressants on signaling from the three subtypes of α1-ARs, namely, α1A-, α1B- and α1D‑ARs, have been little explored. We utilized cell lines overexpressing α1A-, α1B- or α1D-ARs to investigate the effects of the antidepressants imipramine (IMI), desipramine (DMI), mianserin (MIA), reboxetine (REB), citalopram (CIT) and fluoxetine (FLU) on noradrenaline-induced second messenger generation by those receptors. We found similar orders of inhibition at α1A-AR (IMI < DMI < CIT < MIA < REB) and α1D‑AR (IMI = DMI < CIT < MIA), while the α1B-AR subtype was the least engaged subtype and was inhibited with low potency by three drugs (MIA < IMI = DMI). In contrast to their direct antagonistic effects, prolonged incubation with IMI and DMI increased the maximal response of the α1B-AR subtype, and the CIT of both the α1A- and the α1B-ARs. Our data demonstrate a complex, subtype-specific modulation of α1-ARs by antidepressants of different groups.

## 1. Introduction

Depression is a debilitating disorder causing lasting suffering in millions of people, reducing their ability to live normal lives and representing a major, although often hidden, burden to society. It is estimated that more than 300 million people suffer from depression worldwide [1]; for example, in Europe, depression is the most prevalent mental disorder, affecting more than 30 million people each year [2]. The neuronal underpinnings of depression remain elusive; however, clinically, it is commonly treated with drugs modulating monoaminergic neurotransmission [3,4]. It is now apparent that apart from modulating the synaptic levels of serotonin, noradrenaline and dopamine by altering their reuptake or metabolism, many antidepressants can directly bind to the receptors of these monoamines. For example, members of a prototypical class of antidepressants, the tricyclic antidepressants (TCAs), simultaneously inhibit noradrenaline reuptake and antagonize specific noradrenergic receptors, which may lead to self-cancelling actions [3,5,6,7,8]. Conversely, antagonistic properties might shape the actual profile of the receptor activation resulting from elevated noradrenaline levels, potentially contributing to differences in efficacy and side effects.

Physiological responses to noradrenaline and adrenaline are mediated by adrenergic receptors (ARs), which are widely distributed in the central and peripheral nervous systems. These receptors are the seven transmembrane-spanning receptors that belong to the large G-protein-coupled receptor (GPCR) superfamily. The AR family is presently divided into three distinct receptor subclasses, namely, β-, α2- and α1-ARs, and each subclass comprises several subtypes [9]. The α1-AR family consists of three subtypes, which are designated α1A, α1B and α1D [10]. The clone originally called α1C-AR corresponds to the pharmacologically defined α1A-AR [11]. Some tissues possess α1A-ARs that display a relatively low affinity in binding assays for prazosin (the α1-AR antagonist) and are termed α1L-ARs, but recent evidence suggests that they are a phenotypic (conformational) state of α1A-ARs, rather than a distinct entity [12,13,14,15].

α1-ARs function as stimulatory receptors, and each subtype is encoded by a separate gene located on different chromosomes and has a distinct pharmacological profile and amino acid sequence [10,16]. All three α1-AR subtypes, namely, α1A-, α1B- and α1D-ARs, are coupled to Gq/11 and phospholipase Cβ (PLC), which stimulates phosphoinositide hydrolysis to produce two second messengers, diacylglycerol and inositol trisphosphate. This is then followed by protein kinase C (PKC) activation and the increased mobilization of intracellular Ca^2+^. Although the three α1-AR subtypes induce the same cascade of intracellular signaling events, they differ in their subcellular distribution, efficacy in evoking intracellular signals, and transcriptional profiles (see [17,18,19]).

All three α1-AR subtypes are present in the peripheries of both the cardiovascular and genitourinary systems and at relatively high densities throughout the CNS. In the periphery, both the differential distribution and function of the α1A-, α1B- and α1D-AR subtypes are well characterized; for example, this knowledge was applied successfully to minimize the side effects of drugs for benign prostatic hypertrophy [8,19]. However, it has proven much more challenging to understand the functions of α1-AR subtypes in the CNS largely due to a lack of brain-permeable subtype-specific ligands and a lack of specific antibodies [10,18,20,21]. Consequently, while the involvement of bulk α1‑ARs in the pathophysiology of depression and antidepressant action is documented [22,23,24], it is unclear how drug action on different α1-AR subtypes might influence the outcomes of pharmacotherapies for neuropsychiatric disorders. Similarly, the potential for developing drugs specific to either α1A-, α1B- or α1D-ARs in the CNS has not been realized.

More recently, the utilization of genetic mouse models has supported the notion that different α1A-, α1B- and α1D-AR subtypes have not only divergent, but sometimes even opposing, roles in the regulation of behaviors linked with depression and other neuropsychiatric disorders [18,19,22]. There is some evidence that the α1A-AR subtype has trophic-like effects, including increasing neurogenesis and improving mood, while the α1B-AR subtype seems to be required for the behavioral activation elicited by modafinil [25]; however, the overexpression of constitutively active α1B-AR leads to the activation of proapoptotic pathways, suggesting a role in neurodegeneration [26]. Additionally, few studies have utilized in vitro receptor overexpression to assess the potential of specific drugs to act on specific α1-AR subtypes. For instance, drugs belonging to the TCA class, including amitriptyline, nortriptyline and imipramine (which inhibit the noradrenaline transporter), are much weaker antagonists of α1B-AR than of α1A- and α1D-AR [7]. Thus, the authors postulated that α1B-AR activation will be most increased by the augmented noradrenaline availability resulting from the blockade of neuronal reuptake [7]. Recently, Proudman et al. [8] performed complex studies of the subtype selectivity of a wide range of α-antagonists, demonstrating multiple subtype-specific interactions with implications for peripheral side effects. The latter study provided affinity data on binding to the α1A-, α1B- and α1D-AR subtypes for an impressive panel of compounds, demonstrating the strong binding of many clinically relevant antidepressants and antipsychotics to α1-ARs. This supports the relevance of studies of α1-AR subtypes in antidepressant action, and prompts further investigation into the subject. More detailed investigations of specific drugs will be relevant, considering the complex regulation of intracellular signaling from α1-ARs and recent evidence that many compounds can act not only as full antagonists but also as partial or inverse agonists with potentially different short- and long-term effects on α1-AR signaling [27].

Signaling from α1A-, α1B- and α1D-ARs can be regulated at multiple levels, including transcriptional regulation, protein trafficking, and desensitization [18,28]. All three α1-AR subtypes have multiple sites of phosphorylation in their intracellular fragments that play major roles in the regulation of receptor function [19]. Interestingly, these sites are not conserved among subtypes; furthermore, different ligands were shown to elicit distinct phosphorylation patterns at the same receptor [19]. This raises the possibility of differential receptor responses to long-term treatment.

Considering the evidence for the role of α1-ARs in depression and antidepressant action, together with recent data demonstrating the differential binding of clinically relevant antidepressants to the α1A-, α1B- and α1D-AR subtypes, the aim of the current study was to evaluate how the chosen antidepressants modulate intracellular signal transduction from α1A-, α1B- and α1D-ARs, both acutely and after prolonged treatment, by utilizing an in vitro overexpression model and the measurement of second messenger generation.

Our main findings demonstrate that imipramine, desipramine and mianserin directly antagonize the noradrenaline-induced second messenger generation of all three α1-AR subtypes, albeit with different potencies, while reboxetine and citalopram directly antagonized the responses of α1A-ARs and both the α1A- and α1D-AR subtypes, respectively. Importantly, however, we show that prolonged incubation with imipramine and desipramine increases the maximal response to α1B-AR, while citalopram increases the maximal response to the α1A subtype and, to a lesser extent, the α1B subtype. Together, our results show that the interaction of various antidepressants and α1-ARs might involve both the antagonism and the modulation of the strength of intracellular signal transduction.

## 2. Results

### 2.1. Creation and Validation of PC12 Cells Expressing α1A-AR or α1B-AR

**Figure 1:**
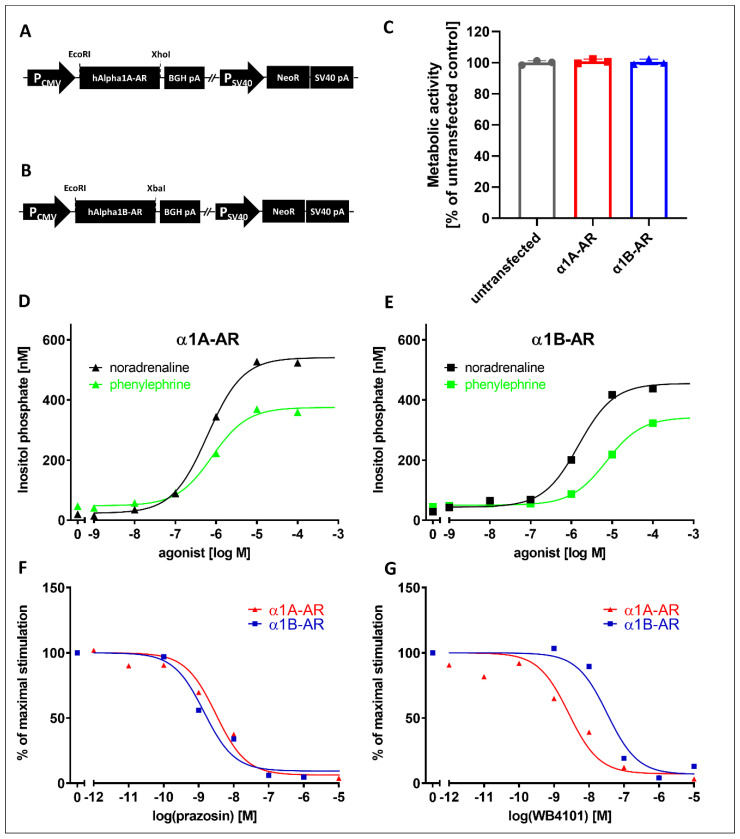
The creation and functional characterization of cell lines stably expressing the α1A- and α1B-adrenergic receptor subtypes in PC12 cells. Expression cassettes of pcDNA3.1+ plasmids used to express human (**A**) α1A- and (**B**) α1B- adrenergic receptors. (**C**) Metabolic activity of untransfected and receptor-expressing lines. Second messenger response of (**D**) α1A- and (**E**) α1B-adrenergic receptor-expressing cells to stimulation with the agonists noradrenaline (black) and phenylephrine (green). Second messenger response to a submaximal (EC_90_) noradrenaline concentration in the presence of the antagonists (**F**) prazosin and (**G**) WB4101. P_CMV_—CMV promoter; hAlpha1A/B-AR—human α1A- and α1B-adrenergic receptors; P_SV40_—SV40 promoter; NeoR—neomycin resistance gene; BGH pA—bovine growth hormone polyA; SV40 pA—SV40 polyadenylation site. Data are shown as the mean ± SEM, *n* = 2–4 wells.

We created two PC12 cell lines stably expressing human α1A-AR or α1B-AR via the transfection of wild-type PC12 cells with pcDNA3.1+ plasmids encoding human α1A-AR and α1B-AR and selection under antibiotics (Figure 1A,B). No adverse effect of receptor expression on cell metabolic activity was detected by resazurin reduction assay (Figure 1C).

The generated cell lines were validated by measuring the response to the agonists noradrenaline and phenylephrine (Figure 1D,E). α1A-AR showed an EC_50_ of 1 × 10^−6.2^ for noradrenaline and an EC_50_ of 1 × 10^−6.1^ (Figure 1D) for phenylephrine, while α1B-AR showed an EC_50_ of 1 × 10^−5.8^ for noradrenaline and an EC_50_ of 1 × 10^−5.1^ for phenylephrine (Figure 1E). The activation of the cloned receptors was blocked by known inhibitors of α1-ARs, prazosin and WB4101. Prazosin inhibited both receptors with similar potencies—IC_50_ = 1 × 10^−8.5^ for α1A-AR and IC_50_ = 1 × 10^−8.8^ for α1B-AR (Figure 1F)—while WB4101 was more potent at inhibiting α1A‑AR (IC_50_ = 1 × 10^−8.6^) than α1B-AR (IC_50_ = 1 × 10^−7.5^) (Figure 1F). For α1D‑AR, we were not able to obtain a PC12 clone with stable functional receptor expression; therefore, commercially available ChemiSCREEN™ Ready-to-Assay™ α1D adrenergic family receptor frozen cells (Millipore, Merck KGaA, Darmstadt, Germany) were used.

### 2.2. Determination of Receptor Binding Sites on Cells

**Figure 2:**
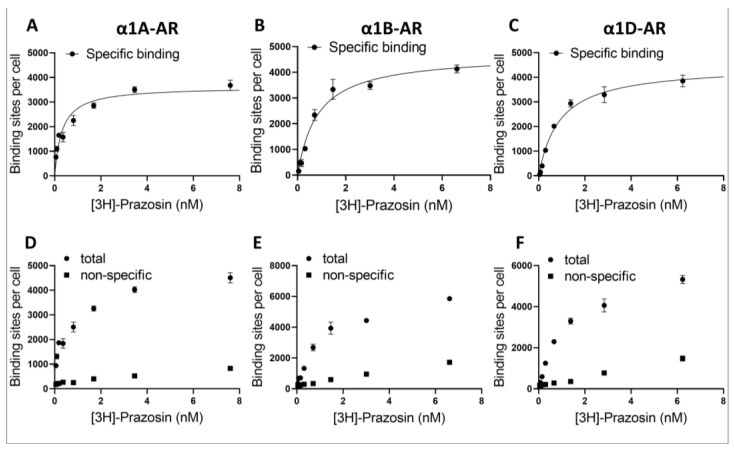
Saturation curves of [^3^H]prazosin binding in the PC-12 cell lines expressing α1A-AR (**A**,**D**) and α1B‑AR (**B**,**E**) and in the ChemiSCREEN™ Ready-to-Assay™ α_1D_ cells (**C**,**F**). Specific binding (**A**–**C**) was determined by subtracting the amount bound in the presence of 10 μM prazosin for [^3^H]prazosin from the total radioactivity bound per milligram protein (**D**–**F**). Each point represents the mean ± SEM of quadruplicate determinations from four experiments.

To determine the number of α1-AR binding sites on the cell surface, saturation binding studies were performed with the labeled α1-AR antagonist [^3^H]prazosin, and in parallel, nonspecific binding was determined in the presence of a non-labeled prazosin (Figure 2).

From the prazosin binding curves, the extrapolated number of maximal binding sites per cell (*B*_max_) was relatively similar for all cells, i.e., 3622 ± 247 binding sites per cell for α1A-AR (Figure 2A), 4694 ± 294 for α1B-AR (Figure 2B) and 4470 ± 240 for α1D-AR (Figure 2C). In these cell lines, the specific [^3^H]-prazosin binding was saturable, reversible and of high affinity with dissociation constants (*K*_D_) of 0.32 ± 0.08, 0.83 ± 0.16 and 0.90 ± 0.14 nM for α1A- (Figure 2A), α1B- (Figure 2B) and α1D-AR (Figure 2C), respectively.

### 2.3. Evaluation of the Antagonistic Effects of Antidepressant Drugs on α1-ARs

**Figure 3:**
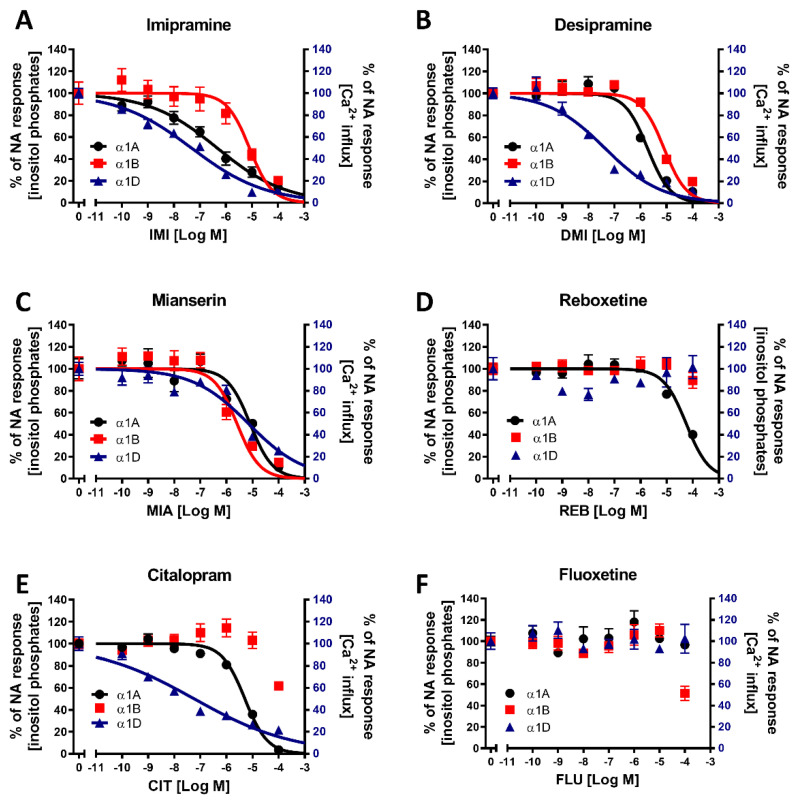
Inhibitory effects of antidepressants on noradrenaline-stimulated second messenger activation. Cells were incubated with varying concentrations of selected antidepressant drugs (imipramine (**A**), desipramine (**B**), reboxetine (**C**), mianserin (**D**), citalopram (**E**), and fluoxetine (**F**)) and noradrenaline at submaximal concentrations (EC_90_). Data are expressed as a percentage of noradrenaline-stimulated second messenger accumulation (inositol phosphate synthesis in PC-12 cell lines expressing α1A- and α1B-AR and calcium ion influx from ChemiSCREEN™ Ready-to-Assay™ α_1D_ cells). Each value represents the mean ± S.E.M. of at least three independent experiments conducted in duplicate.

Inositol phosphate generation was measured as an index of α1A- or α1B-AR reactivity with the use of a TR-FRET-based assay, while in the case of α1D-AR cells, the influx of calcium ions was determined with Fluo-4 dye. To measure the modulatory effects of drugs on the noradrenaline-mediated activation of each of the α1-AR subtypes, cells were pretreated for 10 min with various concentrations of antidepressants before the addition of noradrenaline at a concentration eliciting a submaximal (~EC_90_) receptor response (4 μM for α1A- or α1B-AR and 1 μM for α1D-AR). Then, after 75 or 95 min of incubation (for inositol phosphate or calcium ions measurements, respectively), the levels of second messengers generated were subsequently evaluated. We found that imipramine (Figure 3A), desipramine (Figure 3B), and mianserin (Figure 1C) attenuated the noradrenaline-induced activation of all three α1-AR subtypes, albeit with different potencies (Table 1).

More striking differences in the antagonistic effects of antidepressants between subtypes of α1-ARs were exhibited by reboxetine (Figure 3D), which decreased only the α1A-AR response, and citalopram (Figure 3E), which modulated the activation of α1A- and α1D-ARs but did not demonstrate clear antagonism at the tested doses towards α1B-ARs, having only modest inhibitory effects at the highest measured concentration on this receptor subtype. Additionally, fluoxetine did not exhibit antagonistic properties against any of the α1-ARs in the tested concentration range (Figure 3F), although again, we saw a modest inhibitory effect on α1B-AR at the highest tested concentration.

The order of antidepressant potency in inhibiting α1A-AR was IMI < * DMI < * CIT < * MIA < * REB, which was similar to that of the inhibition of α1D‑AR by these drugs (IMI = DMI < * CIT < * MIA), but the tested drugs had a different order of potency in inhibitting the α1B‑AR subtype (MIA < * IMI = DMI) (* next to < demotes significantly lower IC_50_, *p* < 0.0001).

### 2.4. Effects of Preincubation with Antidepressant Drugs on the Subsequent Activation of α1-ARs by Noradrenaline

**Figure 4:**
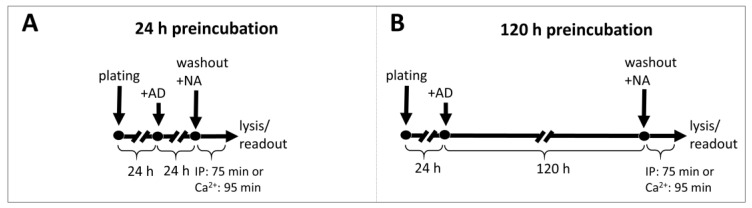
Timelines of experiments testing prolonged (24 h (**A**) and 120 h (**B**)) preincubation of cells expressing α1-AR subtypes with antidepressants. AD—antidepressant drug; NA—noradrenaline.

**Figure 5:**
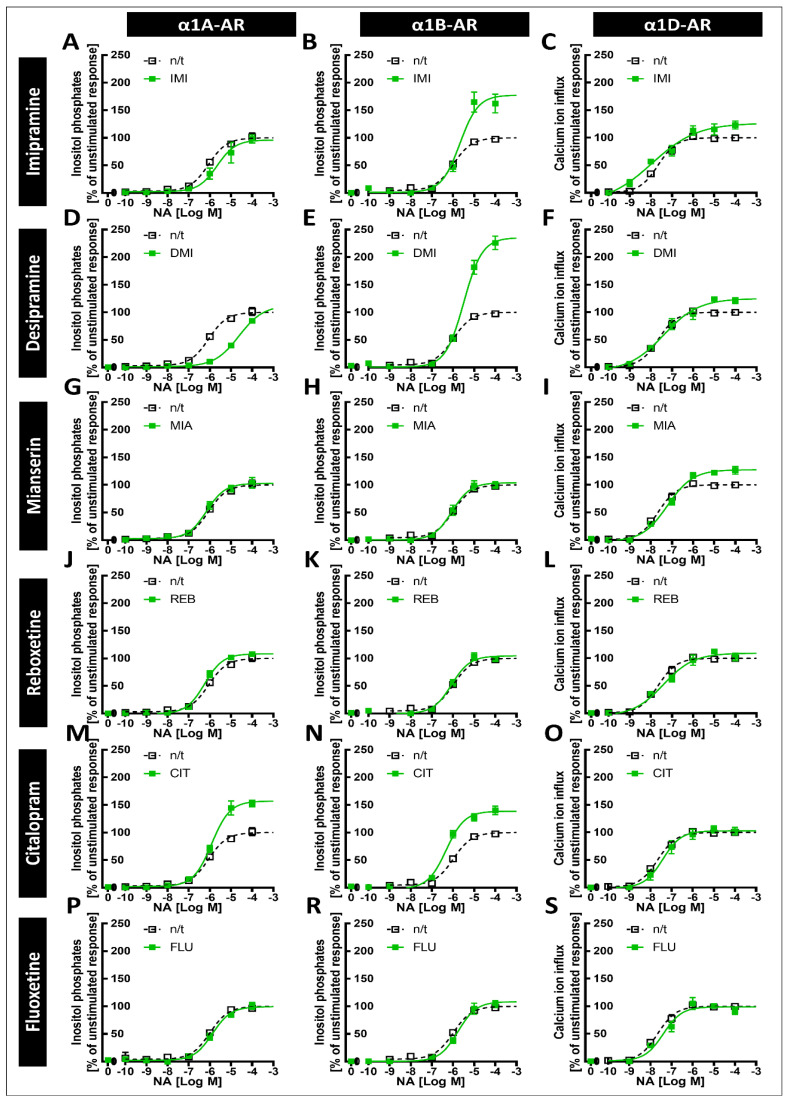
Dose–response curves for noradrenaline and the α1A-, α1B- and α1D-adrenergic receptor subtypes’ second messenger responses after a 24 h preincubation with the chosen antidepressant drugs. The graphs show the responses of cells after preincubation with the antidepressant imipramine (IMI, (**A**–**C**)), desipramine (DMI, (**D**–**F**)), mianserin (MIA, (**G**–**I**)), reboxetine (REB, (**J**–**L**)), citalopram (CIT, (**M**–**O**)) or fluoxetine (FLU, **P**–**S**) (green solid lines on the respective graphs), or the responses of control cells (black dotted lines on all graphs). The effects were tested by measuring the inositol phosphate production by cells expressing α1A-AR (**A**,**D**,**G**,**J**,**M**,**P**) or α1B-AR (**B**,**E**,**H**,**K**,**N**,**R**), or by measuring the noradrenaline-induced calcium ion influx of ChemiSCREEN™ Ready-to-Assay™ α1DA-AR-expressing cells (**C**,**F**,**I**,**L**,**O**,**S**). Each value represents the mean ± S.E.M. of at least four independent experiments conducted in duplicate.

**Figure 6:**
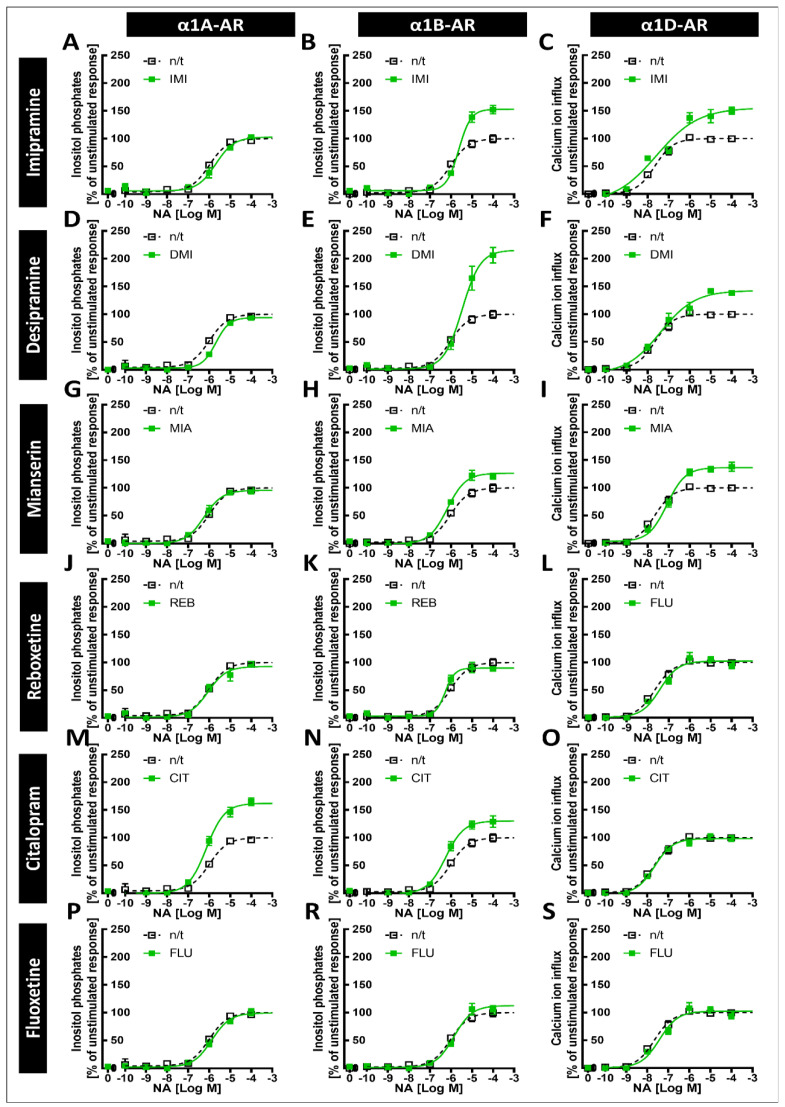
Dose–response curves for noradrenaline and the α1A-, α1B- and α1D-adrenergic receptor subtypes’ second messenger responses after a 120 h preincubation with the chosen antidepressant drugs. The graphs show the responses of cells after preincubation with the antidepressant imipramine (IMI, (**A**–**C**)), desipramine (DMI, (**D**–**F**)), mianserin (MIA, (**G**–**I**)), reboxetine (REB, (**J**–**L**)), citalopram (CIT, (**M**–**O**)) or fluoxetine (FLU, (**P**–**S**)) (green solid lines on the respective graphs), or the responses of control cells (black dotted lines on all graphs). The effects were tested by measuring inositol phosphate production by cells expressing α1A-AR (**A**,**D**,**G**,**J**,**M**,**P**) or α1B-AR (**B**,**E**,**H**,**K**,**N**,**R**), or by measuring the noradrenaline-induced calcium ion influx of ChemiSCREEN™ Ready-to-Assay™ α1DA-AR-expressing cells (**C**,**F**,**I**,**L**,**O**,**S**). Each value represents the mean ± S.E.M. of at least four independent experiments conducted in duplicate.

To determine whether prolonged incubation with antidepressant drugs can modulate signaling from different α1-AR subtypes, we cultured the cells in the presence of selected antidepressants (10 µM) for 24 h (Figure 4A and Figure 5, Table 2) or 120 h (Figure 4B and Figure 6, Table 3).

**Figure 7:**
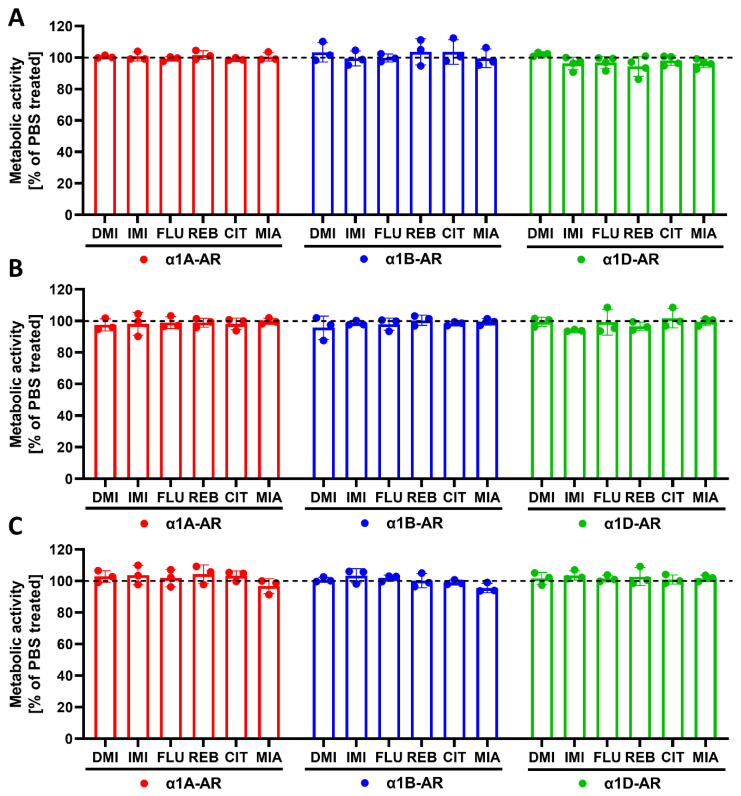
Effects of incubation with the chosen antidepressants on the metabolic activity of cells expressing the α1A-, α1B- or α1D- adrenergic receptor subtypes. The cells were incubated with the chosen antidepressants for 30 min (**A**), 24 h (**B**) or 120 h (**C**). IMI—imipramine; DMI—desipramine; MIA—mianserin; REB—reboxetine; CIT—citalopram; FLU—fluoxetine or PBS (dashed line). Data are shown as the mean ± SD; *n* = 3–4 independent experiments.

After incubation, the cells were harvested, the antidepressants were washed out, and the cells were stimulated for 75 or 95 min (for inositol phosphates or calcium ions measurements, respectively) with noradrenaline at concentrations ranging from 1 × 10^−10^ to 1 × 10^−4^ M, followed by the measurement of second messenger generation. We found subtype-specific differences in the response to noradrenaline after preincubation with different antidepressant drugs. Namely, imipramine increased the maximal response of α1B-AR after 24 h of preincubation (Figure 5B, Table 2). The effect of imipramine on the maximal response of α1B-AR was maintained after 120 h of incubation (Figure 6B, Table 3); additionally, after 120 h, imipramine also significantly increased the maximal response of α1D-AR and slightly shifted the EC_50_ for both of these receptors (Figure 6B,C, Table 3). Desipramine had similar, but even more pronounced, effects to imipramine on α1B- and α1D-AR (Figure 5E and Figure 6E,F, Table 2 and Table 3); however, it additionally shifted the EC_50_ of α1A-AR after 24 h (Figure 5D, Table 2) but not 120 h (Figure 6D, Table 3). Mianserin had no effect after 24 h (Figure 5G–I, Table 2), but slightly increased the maximal responses of α1B- and α1D-ARs after 120 h (Figure 6G–I, Table 3), and shifted the EC_50_ of the latter (Figure 6I, Table 3). Citalopram, in contrast to imipramine, desipramine and mianserin, which acted predominantly on α1B- and α1D‑ARs, only had an effect on α1A- and α1B-ARs, increasing their maximal response with a more pronounced effect on the former after both 24 h (Figure 5M,N, Table 2) and 120 h (Figure 6M,N, Table 3), while not modifying their EC_50_. Preincubation with reboxetine or fluoxetine had no effect on any of the α1-ARs (Figure 5J–L,P–S and Figure 6J–L,P–S, Table 2 and Table 3). Preincubation with antidepressants had no effect on metabolic activity as measured by the resazurin reduction assay (Figure 7).

## 3. Discussion

Over the last 30 years, it has become clear that α_1_-ARs are not a homogenous group and consist of three subtypes (see [29]). While older studies have proven that clinically relevant antidepressants either directly antagonize α1-ARs [6,30,31] or can influence the strength of their intracellular signaling [6,32], they have not established clearly whether these effects vary between α1A-, α1B- and α1D-ARs. In contrast to the physiological functions of α1‑AR subtypes in peripheral tissues, which have been relatively well studied and selectively engaged pharmacologically, only a few recent studies have more consistently investigated the effects of different antidepressant and antipsychotic drugs on α1A-, α1B- and α1D-ARs [7,8]. The different functions of distinct α1-AR subtypes in the CNS were recognized only recently with the use of genetic animal models that demonstrated their distinct roles in the regulation of behavior [18], raising the possibility that the action of antidepressants on α1A-, α1B- and α1D‑ARs might be relevant not only to their possible peripheral side effects but also to modulating the therapeutic outcomes of treatments with those drugs. In recent years, it has also become apparent that the three α1-AR subtypes differ not only in ligand binding potency but also in that their signaling strength can be differentially regulated [12,19]. Altogether, there is a clear need to further investigate the effects of antidepressants on signaling through specific α1-AR subtypes, which might hold promise for the future design of drugs that are both more tolerable and more effective. In the present manuscript, we investigated the possible differences in the susceptibility of α1-AR subtypes to regulation by antidepressant drugs, both in acute and long‑term treatment.

To study the effects of the drugs on intracellular signaling from α1-AR subtypes, we utilized cell lines stably expressing those receptors. We created cells stably expressing the α1A- and α1B-AR subtypes. These cell lines demonstrated the expected responses to known α1A- and α1B-AR ligands. Both receptors showed similar activation by noradrenaline and phenylephrine, and as expected, prazosin demonstrated identical inhibitory effects on both receptors, while inhibition with WB4101 clearly distinguished α1A- and α1B-subtypes, similarly to other studies [23,24,33,34]. Together with the lack of differences in the metabolic activity of cells overexpressing α1A- or α1B-ARs, these results demonstrate their suitability for pharmacological studies. Unfortunately, we were not able to obtain functionally active α1D-AR clones. This is not entirely surprising, as other studies have demonstrated that overexpressed α1D-AR does not seem to properly reach the cell surface unless its N-terminal region is truncated [35] or it is expressed together with α1B-AR [36]. Interestingly, it was recently proposed that α1D-AR can be physiologically cleaved by removing 91 N-terminal amino acids with an unknown protease, suggesting that this truncation might play a role in the regulation of receptor expression [37]. In a recent study investigating the binding of different antidepressants and antipsychotics, Proudman et el. [8] utilized a full-length receptor; however, the surface expression of α1D-AR was 3–10 times lower than that of the α1A- and α1B-AR subtypes, which was still usable for binding studies, in spite of the low signal-to-noise ratio. However, for functional studies, we decided to utilize a commercially available cell line overexpressing functionally truncated α1D-AR (see Section 4).

The utilization of different expression systems for α1D-AR was perhaps the biggest limitation of our study, and the proper interpretation of the data from α1D-AR requires special consideration. We have established that the utilized α1D-AR cell line exhibited a similar number of surface binding sites to those in our α1A- and α1B-AR-overexpressing lines, as demonstrated by prazosin binding. Nonetheless, despite similar surface receptor numbers, it is important to consider that α1D-ARs were expressed in Chem-1 cell lines, while α1A- and α1B-AR were in PC12 cells. Different expression systems might influence intracellular signaling from GPCRs, for example due to the different availability of intracellular signaling cascade components [27]. In fact, the Chemi-1 cells used to study α1D-ARs express high levels of G protein Gα15, which strongly couples expressed α1D-ARs to calcium signaling. Consequently, in studying α1D‑ARs, we measured the intracellular calcium mobilization as a measure of receptor activity. Altogether, this warrants caution when directly comparing between α1A- and α1B-ARs and α1D-ARs. The utilization of calcium assay for the measurement of α1D-ARs activation could explain the different shapes of dose–response curves we observed for α1D-ARs when testing the direct antagonistic action of antidepressants. This could be caused, for example, by the ability of antidepressants to modulate calcium signaling downstream of the phospholipase C/inositol phosphates pathway. However, since it is currently unclear what the most relevant physiological form of surface-expressed α1D‑ARs (truncated, full length, heterodimer or another form) is in the CNS, a direct comparison between α1A- and α1B-AR vs. α1D-ARs IC_50_ values would have to be done cautiously regardless of the model used. Future studies will hopefully be able to investigate multiple second messenger pathways simultaneously for all α1-AR subtypes in order to take into account biased signaling, and do it in multiple cellular systems, which might influence the antagonistic properties of ligands [27]. To make matters more complicated, different α1-AR subtypes can be co-expressed in the same cell type and are known to interact—in fact, α1B-AR and α1D-AR co-expression can rescue the membrane localization of the latter [36]. Moreover, it is now accepted that GPCR can interact either directly or through the interaction of their intercellular partners with other GPCRs, receptor tyrosine kinases, steroid hormone, and other receptors, which altogether creates an incredibly convoluted signaling landscape. Complex interactions within the intracellular signaling pathways probably explain the cell-specific and sometimes seemingly contradicting functions of receptors that seemingly activate the same intracellular components, such as α1A- and α1B-ARs [18]. Our study indicates that different antidepressants will modulate these complex signaling networks trough interaction with different subtypes of α1-ARs. However, a full understanding of the physiological outcome of antidepressant action on α1-AR subtypes will require investigations of not only specific α1-AR subtypes, but also their interactions with other receptors, in the context of specific intracellular environments.

The tricyclic antidepressants IMI and DMI showed antagonistic effects on all three subtypes of α1-ARs, with the lowest affinity for α1B-AR. Our results for imipramine are similar to those of Nojimoto et al. [7], suggesting that the least inhibited α1B-AR might actually be the receptor whose activation is the strongest, considering the global increase in noradrenaline release caused by IMI. Interestingly, while Nojimoto et al. [7] showed similar profiles for amitriptyline and nortriptyline, we observed these for another tricyclic antidepressant, DMI, suggesting that other drugs of this class might cause the strong inhibition of α1A- and α1D-ARs, and the relatively weak inhibition of the α1B-AR subtype. Indeed, Proudman et al. [8] very recently screened a total of 11 tricyclic antidepressants for their binding to α1-AR subtypes, and all of the studied drugs showed the strongest affinity for α1A-AR. They demonstrated that IMI binds with more potency to α1A-AR than to DMI, which is also seen in the significantly lower IC_50_ of IMI than DMI for the inhibition of α1A-AR in our study. Interestingly, Proudman et al. [8] showed a similarly lower Kd for IMI in both the α1B-AR and α1D-AR subtypes, while both we and Nojimoto et al. [7] observed the stronger inhibition of α1A-AR and α1D-AR, with lower potency against the α1B-AR subtype only. Both we and Nojimoto et al. [7] utilized truncated α1D-ARs, raising the possibility that this might be the cause of discrepancies with the study by Proudman et al. [8], in which full-length receptors, albeit expressed at relatively low levels, were studied. Interestingly, the lower potency of IMI to inhibit α1B-AR, but not α1D-AR or α1A-AR, was confirmed in tissue preparations [7], which supports the hypothesis that in at least some tissues, the truncated form of α1D-AR might be physiological, as was proposed after the discovery of endogenously cleaved α1D-AR [37]. Truncated α1D-ARs have been shown to have different affinities for known agonists, further suggesting the possibility that they might also have different affinities for antidepressants. Nonetheless, while this is an interesting possibility, we have not aimed to explore pharmacological differences between different α1D-AR forms, and our data only suggest that this topic should be explored in further studies. We have seen a similar, albeit weaker, inhibition of α1-ARs by CIT, with the weakest effect on α1B-AR, and a very modest inhibition of the α1A-AR subtype, similarly to what was shown by Proudman et al. [8]; again, however, while the other group observed an even weaker inhibition of α1D-AR than α1A-AR, we observed the opposite. We also observed the inhibition of all three subtypes by the tetracyclic antidepressant MIA, and the very weak antagonistic effects of REB towards α1A-AR only and FLU towards α1B-AR.

Our previous studies in animals suggested that exposure to antidepressants might influence long-term signaling from α1-ARs [6,32], while the current data show that antidepressants can directly exert such effects on specific α1-AR subtypes. Perhaps most interestingly, both IMI and DMI, which bind to all three subtypes but inhibit α1B-AR with the lowest potency, considerably increase the maximal response evoked by this receptor. This effect was present after both 24 h and 120 h of preincubation, and was not dependent on changes in cell metabolism, as we did not observe the modulation of metabolic activity by any of the tested drugs. Interestingly, there was no increase in the maximal response of α1A-AR after preincubation with IMI or DMI, suggesting that these changes depend directly on receptor properties rather than changes in intracellular signaling components, as in such cases one would expect to observe an effect for both α1A-AR and α1B-AR, which were expressed in the same cell type. We also observed a smaller but still significant increase in the maximal response of α1D-AR after 120 h, but not 24 h, of incubation with IMI and DMI. It was already proposed by Nojimoto et al. [7] that due to the inhibition of α1A-AR and α1D-AR, the weaker inhibition of α1B-AR, and the increase in noradrenergic tone, the net result of IMI would be increased signaling, specifically through α1B‑AR. Our data support their findings, extend them to desipramine, and furthermore show that the increase in α1B-AR activity might be much more pronounced due to the increased maximal response. Additionally, incubation with desipramine for 24 h caused an EC_50_ shift; however, this effect did not persist after incubation for 120 h.

CIT caused an increase in the maximal response of α1A-ARs to noradrenergic stimulation, and a smaller but still significant increase in the maximal response of α1B-ARs. This is in line with our previous work, where we observed an increase in the maximal α1-AR response to noradrenaline after preincubation with CIT in brain tissue samples [32]. In this earlier study, we demonstrated that CIT attenuated the desensitization of α1-ARs by protein kinase C (PKC) [32]. PKC can desensitize α1-ARs both homo- and heterologously; therefore, it could not be ruled out that the effects did not occur via direct interaction with α1-ARs. Intriguingly, in the current study, the effect of CIT on the maximal receptor response was significantly greater for α1A-AR than for α1B-AR, which reflects the greater modulatory effect of CIT on α1A-AR and its much lower affinity for α1B-AR; however, no effect on the maximal response to noradrenaline was seen for α1D-AR, despite the inhibitory effects of CIT on the activation of this subtype. Nonetheless, the greater long-term effect on α1A-AR than on α1B-AR, which corresponded with the affinities of CIT to those receptors, suggests that the effects of CIT are dependent on its direct effects on α1-AR subtypes. During preincubation with antidepressants, no agonist was present; therefore, the drugs might actually inhibit the intrinsic activity of the receptors. Inverse agonism has been demonstrated now to be common rather than an exception, including for α1-AR ligands [27]. Actually, α1A-AR has been shown to be internalized in an agonist-independent way, and this was linked with constitutive activity [38]. Therefore, it is tempting to speculate that the observed effects of CIT might be linked with the inverse agonism of α1A- and α1B-ARs. However, the picture is certainly complex, since we have also observed that MIA increases the maximal responses of α1B- and α1D-ARs, but not α1A-ARs, after 120 h of incubation, while it has similar potency for the inhibition of all three α1-AR subtypes, with a similar order of magnitude as CIT in its effect on α1A-ARs. The data on MIA are corroborated by our older studies, where we demonstrated that MIA does increase the maximal response of α1-ARs to noradrenaline in brain tissue [6]. Therefore, our in vitro system replicated data from studies in brain tissue, supporting their validity.

Taken together, our data confirm the subtype-specific inhibition of α1-ARs by many clinically relevant antidepressants at the functional level. Furthermore, our results demonstrate that these drugs can modulate α1‑AR function in complex and subtype-specific ways, which is in line with the multiple ways these receptors can be regulated [12,19]. Specifically, our data support the notion that TCAs affect α1B-AR signaling and suggest a possible role of α1A-AR in the action of CIT. The functional importance of this phenomenon needs to be further explored.

Further studies will be needed to uncover the mechanisms regulating α1-AR responses, and it would be beneficial if such studies could be performed in neuronal systems. Moreover, further studies in animals, for example studies utilizing-subtype selective knockouts, are necessary to uncover the impact of the described interactions of antidepressants with specific α1-AR subtypes on the behavioral outcomes of antidepressant therapies.

## 4. Materials and Methods

### 4.1. Cell Culture and Transfection

The rat PC12 pheochromocytoma cell line was obtained from the DSMZ repository. The cells were cultured at 37 °C in a humidified incubator with 5% CO_2_ atmosphere in cell culture dishes coated with collagen type I (Corning BioCoat, Corning, NY, USA). The cell culture medium consisted of RPMI 1640 medium supplemented with 10% horse serum, 5% fetal bovine serum, 10 mg/mL streptomycin, 100 U/mL penicillin and selection antibiotic for stably transfected cells. pcDNA3.1+ plasmids expressing human α1A-AR and α1B-AR were obtained from the Missouri S&T cDNA Resource Center (#AR0A1A0001 and #AR0A1B0000). Cell transfection was performed with Lipofectamine 2000 reagent (Invitrogen/Thermo Fisher Scientific, Waltham, MA, USA) according to the manufacturer’s instructions. Stably transfected cells were selected by G418 (500 µg/mL, Sigma) and assayed for the functional activity of their receptors. To assess the activity of α1D-AR, we used ChemiSCREEN™ Ready-to-Assay™ α1D adrenergic family receptor frozen cells (MilliporeSigma, Saint Louis, MO, USA) and a Fluo-4 Direct™ calcium assay kit (Thermo Fisher Scientific, Waltham, MA, USA).

### 4.2. IP Accumulation Assay

The IP accumulation assay was performed with the IP-One HTRF^®^ assay kit (PerkinElmer/Cisbio, Waltham, MA, USA). Cells with stable expressions of α1A- or α1B-AR were removed with a mix of PBS-Versene (Gibco/Thermo Fisher Scientific, Waltham, MA, USA) (1:1), centrifuged for 5 min (200× *g*) and suspended in stimulation buffer (HEPES 10 mM, CaCl_2_ 1 mM, MgCl_2_ 0.5 mM, KCl 4.2 mM, NaCl 146 mM, glucose 5.5 mM, LiCl 50 mM, pH 7). A total of 14,000 cells resuspended in 7 µL were seeded in each well of a 384-well plate. The next steps were performed according to the manufacturer’s protocol.

(1) Noradrenaline or other agonist stimulation: 7 μL of consecutive concentrations of agonist in stimulation buffer was added to each well.
(2) Antagonistic properties of drugs: 3.5 μL of various concentrations (from 4 × 10^−10^ to 4 × 10^−4^ M) of drug in stimulation buffer were added to each well, and after 5 min, 3.5 μL of noradrenaline was added for a final concentration of 4 μM (EC_90_).

After 75 min of incubation at 37 °C, the detection reagent was added, and after 2 h of incubation at room temperature, the time-resolved fluorescence at wavelengths of 620 nm and 655 nm was measured. IP One concentration was calculated according to the manufacturer’s instructions. The results are presented as percentages of the maximal (agonistic stimulation measurement) or EC_90_ (antagonistic property measurement) response.

### 4.3. Calcium Assay

ChemiSCREEN™ Ready-to-Assay™ α1D adrenergic family receptor frozen cells (MilliporeSigma, Saint Louis, MO, USA) were cultured in DMEM (Gibco/Thermo Fisher Scientific, Waltham, MA, USA) supplemented with 10% FBS. They were removed with trypsin/EDTA (Gibco/Thermo Fisher Scientific, Waltham, MA, USA) and resuspended in assay buffer (30 mL of 1 M HEPES to 1.47 L of 1X HBSS, pH 7.3).

A Fluo-4 Direct™ calcium assay kit (Thermo Fisher Scientific, Waltham, MA, USA) was used per the manufacturer’s instructions. Briefly, cells were plated in 5 µL of assay buffer at a density of 17,000 cells/well in standard tissue culture-grade, clear-bottom, black 384-well plates. The cells were incubated for 1 h at 37 °C, and the test compounds were added to the cells.

(1) Stimulation with noradrenaline: 5 μL of consecutive concentrations of noradrenaline in stimulation buffer was added to each well.
(2) Antagonistic properties of drugs: 2.5 μL of various concentrations (from 4 × 10^−10^ to 4 × 10^−4^ M) of drug in stimulation buffer were added to each well, and after 5 min, 2.5 μL of 1 μM NE (EC_90_) was added.

After 5 min, 10 µL of the 2X Fluo-4 Direct™ calcium reagent loading solution was loaded into the cells for 30 min at 37 °C and 1 h at room temperature. The fluorescence was measured with excitation at 494 nm and emission at 516 nm. The calcium ion concentration was calculated according to the manufacturer’s instructions. The results are presented as percentages of the maximal (agonistic stimulation measurement) or EC_90_ (antagonistic property measurement) response.

### 4.4. Preincubation with Antidepressants

Cells were seeded at 60% confluence (for 24 h incubation) or at 25% confluence (for 5-day incubation) in standard growth medium with 10 μM antidepressant or vehicle. After 24 h or 120 h, the cells were washed and harvested for IP1 or calcium ions measurement after stimulation with NA as described above.

All experiments were performed in duplicate and repeated at least 4 times.

### 4.5. Binding Assay

The PC12 cells with stable expressions of α1A- or α1B-AR and ChemiSCREEN™ Ready-to-Assay™ α1D cells were washed and resuspended in 50 mM Tris-HCl, pH 7.4 buffer, followed by disruption in the tissue homogenizer Polytron and centrifugation for 45 min (35,000× *g*) at 4 °C. The supernatant was removed and homogenization and centrifugation steps were repeated. Obtained pellets were dissolved in buffer and frozen at −80 °C. Subsequent measurements were performed in 96 well-plates. For total binding, 25 µL of buffer (20 mmol/L Tris, 150 mmol/L NaCl, 1 mmol/L MgCl_2_, EDTA 1 mM pH 7.4), 25 µL of 8 [^3^H]-Prazosin (Perkin–Elmer) concentration (from 0.125 to 16 nM) and 150 µL of diluted pellet were used. For nonspecific binding, the buffer was replaced with 25 µL of 10 µM Prazosin (Sigma). After 1 h of incubation with shaking at 37 °C, the probes were harvested into 96-wells plates with GF/B filter (Perkin-Elmer) and washed 4 times. Subsequently, after overnight incubation in the dark, 35 µL of scintillate (Ultima Gold LSC Cocktail Sigma-Aldrich, Saint Louis, MO, USA) was added, and luminescence was measured. The protein concentration in pellet form was diluted with the Lowry method. The protein concentrations were transformed into cell numbers from experimentally calculated formula, whereby 0.25 mg of protein comes from 1.1 million cells. Experiments were conducted 4 times in tetraplicates.

### 4.6. Compounds

DL-Norepinephrine hydrochloride (A7256), citalopram hydrobromide (C7861), desipramine hydrochloride (D3900), mianserin (M2525) and imipramine hydrochloride (I7379) were purchased from Sigma-Aldrich (St. Louis, MO, USA). Fluoxetine hydrochloride (0927) and reboxetine mesylate (1982) were purchased from Tocris Bioscience (Bristol, UK).

### 4.7. Data Processing and Statistical Analysis

The analysis was performed with GraphPad Prism 5.0 (binding assay) or GraphPad Prism 9.0 (the remaining experiments):

(1) The one-site specific binding curves were fitted to total nonspecific binding curves, and the Kd and Bmax parameters were calculated;
(2) The agonist dose–response curves were fitted with 3-parameter (log(agonist) vs. response) and 4-parameter (log(agonist) vs. response–variable slope) models. Model accuracy was compared with the F test, and a simpler (3-parameter) model was chosen unless a significance threshold of *p* < 0.05 was reached, in which case a 4-parameter model was chosen;
(3) For fitting the inhibitory effects, the dose–response curves were fitted with 3-parameter (log(inhibitor) vs. normalized response) and 4-parameter (log(inhibitor) vs. normalized response-variable slope) models. Model accuracy was compared with the F test, and a simpler (3-parameter) model was chosen unless a significance threshold of *p* < 0.05 was reached, in which case a 4-parameter model was chosen.

Statistical differences between logIC_50_ values were calculated with the multiple *t* test with the Holm–Šidák correction. The logEC_50_ and maximal response values were compared with 2-way ANOVA with the Holm–Šidák multiple comparison test.

### 4.8. Metabolic Activity Assay

To exclude the possible adverse effects of drugs on cells, metabolic activity was measured. The cells were seeded on 96-well plates at densities of 40,000 cells/well (for 30 min and 24 h incubations) or 20,000 cells/well per well (for 120 h incubation) for α1A- or α1B-AR-expressing cells, or at densities of 20,000 cells/well (for 30 min and 24 h incubations) or 10,000 cells/well (for 120 h incubation) for α1D-expressing cells. The cells were incubated with antidepressants at a 10 µM concentration for the indicated times. Metabolic activity was measured with a resazurin assay (Sigma-Aldrich, St. Louis, MO, USA) according to the manufacturer’s protocol.

## Figures and Tables

**Table 1:** The effects of drugs in cells with cloned α1-AR subtypes. The IC_50_ values were determined by nonlinear regression analysis from the dose–response curves in the cells expressing each subtype of α1-AR after stimulation with noradrenaline at the EC_90_ concentration. Each value is the mean and SEM from at least three independent experiments in duplicate. IMI—imipramine; DMI—desipramine; MIA—mianserin; REB—reboxetine; CIT—citalopram; FLU—fluoxetine.

	α1A	α1B	α1D
Drug	logIC_50_ (M)	logIC_50_ (M)	logIC_50_ (M)
IMI	−6.33 ± 0.14	−5.09 ± 0.13	−7.3 ± 0.09
DMI	−5.73 ± 0.08	−5.1 ± 0.06	−7.36 ± 0.13
MIA	−5.09 ± 0.12	−5.59 ± 0.11	−5.15 ± 0.15
REB	−4.25 ± 0.09	n.a.	n.a.
CIT	−5.29 ± 0.04	>−4	−7.23 ± 0.13
FLU	n.a.	>−4	n.a.

**Table 2:** Effects of 24 h preincubation with chosen antidepressants on the EC_50_ and maximal response (MAX) of the second messenger response to noradrenaline in the α1A-, α1B- and α1D-adrenergic receptor subtypes. n/t—nontreated control; IMI—imipramine; DMI—desipramine; MIA—mianserin; REB—reboxetine; CIT—citalopram; FLU—fluoxetine. ** *p* < 0.01, *** *p* < 0.001 vs. the n/t group, ^###^ *p* < 0.01 vs. the α1A group.

	α1A		α1B		α1D	
	EC_50_ (logM)	MAX	EC_50_ (logM)	MAX	EC_50_ (logM)	MAX
n/t	−6.1 ± 0.1	100 ± 3	−6 ± 0.1	100 ± 2	−7.7 ± 0.1	100 ± 2
IMI	−5.7 ± 0.2	96 ± 8	−5.7 ± 0.1	177 ± 11 ***	−7.9 ± 0.4	127 ± 9
DMI	−4.6 ± 0.2 ***	113 ± 12	−5.5 ± 0.1 **	235 ± 8 ***	−7.4 ± 0.2	125 ± 7
MIA	−6.1 ± 0.1	103 ± 4	−6 ± 0.1	104 ± 4	−7.2 ± 0.1	127 ± 4
REB	−6.2 ± 0.1	108 ± 3	−6.1 ± 0.1	105 ± 4	−7.4 ± 0.2	109 ± 5
CIT	−5.9 ± 0.1	157 ± 5 ***	−6.3 ± 0.1	139 ± 4 *** ^###^	−7.4 ± 0.1	103 ± 4
FLU	−5.9 ± 0.2	93 ± 6	−5.8 ± 0.1	108 ± 5	−7.4 ± 0.1	99 ± 4

**Table 3:** Effects of 120 h preincubation with the chosen antidepressants on the EC_50_ and the maximal response (MAX) of the second messengers to noradrenaline for the α1A-, α1B- and α1D-adrenergic receptor subtypes. n/t—nontreated control; IMI—imipramine; DMI—desipramine; MIA—mianserin; REB—reboxetine; CIT—citalopram; FLU—fluoxetine. * *p* < 0.05, ** *p* < 0.01, *** *p* < 0.001 vs. the n/t group, ^###^ *p* < 0.01 vs. the α1A group.

	α1A		α1B		α1D	
	EC_50_ nM	MAX	EC_50_ nM	MAX	EC_50_ nM	MAX
n/t	−6.0 ± 0.1	100 ± 3	−6.0 ± 0.1	100 ± 4	−7.7 ± 0.1	100 ± 2
IMI	−5.7 ± 0.1	103 ± 5	−5.6 ± 0.1 **	153 ± 6 ***	−7.2 ± 0.1 *	155 ± 10 ***
DMI	−5.7 ± 0.1	94 ± 3	−5.5 ± 0.1 ***	215 ± 12 ***	−7.4 ± 0,2	143 ± 7 ***
MIA	−6.2 ± 0.1	96 ± 3	−6.2 ± 0.1	127 ± 4 **	−7.1 ± 0.1 ***	137 ± 3 ***
REB	−6.3 ± 0.1	93 ± 5	−6.3 ± 0.1	90 ± 4	−7.4 ± 0.1	104 ± 4
CIT	−6.1 ± 0.1	162 ± 5 ***	−6.3 ± 0.1	130 ± 5 *** ^###^	−7.7 ± 0.1	98 ± 2
FLU	−5.9 ± 0.1	99 ± 4	−5.9 ± 0.1	113 ± 5	−7.4 ± 0.1	102 ± 3

## Data Availability

The data presented in this study are available upon reasonable request from the corresponding author.

## Abbreviations

AD	antidepressant drug
AR	adrenergic receptor
CNS	central nervous system
GPCR	G-protein-coupled receptor
NA	noradrenaline
PKC	protein kinase C
PLC	phospholipase C
TCA	tricyclic antidepressant drug
